# Adrenal Crisis During Cesarean Section in a Patient With Primary Adrenal Insufficiency

**DOI:** 10.7759/cureus.49964

**Published:** 2023-12-05

**Authors:** Inês Pestana, Henrique Guimarães, Alexandra Saraiva, Dalila Veiga, Humberto Machado

**Affiliations:** 1 Anesthesiology, Centro Hospitalar Universitário de Santo António, Porto, PRT

**Keywords:** obstetric anesthesia, life-threatening emergency, cardiovascular collapse, adrenal crisis, primary adrenal insufficiency, congenital adrenal hyperplasia

## Abstract

Congenital adrenal hyperplasia (CAH) is a type of primary adrenal insufficiency (AI) that predisposes to adrenal crisis (AC) during stress. We describe a case of a primipara with CAH who was admitted in labor. To prevent AC, glucocorticoid replacement was given according to guidelines. Due to fetal decelerations, an emergency C-section was performed under general anesthesia following which refractory hypotension emerged. The diagnosis of AC was considered, and hydrocortisone was given with sustained hemodynamic improvement. AC is a life-threatening emergency whose diagnosis requires a high index of suspicion. Despite adequate steroid coverage, additional stressors may precipitate AC, so it is of paramount importance that anesthesiologists consider this emergency.

## Introduction

Cortisol is the dominant glucocorticoid in humans and the primary hormone involved in the physiological stress response [1]. It is produced in the zona fasciculata of the adrenal cortex and released in a circadian rhythm. Daily production is up to 20 mg each day and increases following surgery (and other causes of physiological stress), proportionally along with proinflammatory cytokines, and returns to baseline values within 24-48 hours [2]. A five-fold increase in cortisol levels is common during major surgery under general anesthesia. During labor, cortisol levels also rise due to physiological stress and pain related to uterine contractions and cervical dilatation, irrespective of the analgesia technique [3]. 

Primary adrenal insufficiency (PAI) relates to conditions where the underlying cause lies within the adrenal gland, including auto-immune (Addison’s disease) and congenital causes (congenital adrenal hyperplasia (CAH)), the latter has an estimated prevalence of 1 in 15000 births and is treated with chronic glucocorticoid replacement [2]. This condition predisposes to adrenal crisis (AC) during stress periods. All steroid-dependent patients are at risk of adrenal crisis, however, in contrast to secondary adrenal insufficiency, PAI is characterized by insufficient secretion of both cortisol and aldosterone [2].

Insufficient cortisol production during a surgical stress response leads to progressive loss of vasomotor tone, leading to orthostatic hypotension, supine hypotension, and finally, shock, which will be fatal if not rapidly corrected. The cardinal sign of acute adrenal insufficiency, severe hypotension resistant to both volume expansion and vasopressors, may be a late or even agonal event [2].

Patients with AI at labor are at risk for acute adrenal insufficiency. Adrenal crisis in pregnancy can cause severe hypotension, which may affect placental perfusion and therefore, fetal wellbeing. This condition requires immediate treatment as it can be associated with significant maternal and fetal morbidity and mortality if untreated or undiagnosed [4].

It is of paramount importance that replacement therapy is not interrupted, and that the daily dose is increased at times of physiological stress. The recommendations for peri-and postoperative surgical stress doses are the same for pregnancy as for other adults. During delivery, 100 mg hydrocortisone should be administered at the onset of active labor, followed by an infusion of 200 mg in 24 h or 50 mg 6/6h with rapid tapering over 1-3 days to the regular replacement dose after an uncomplicated delivery [2]. Hydrocortisone is the drug of choice due to its pharmacological and pharmacokinetic features, namely its fast onset of action, inability to cross placental tissue, and also because of its mineralo- and glucocorticoid activity [2,4].

Perioperative Addisonian crisis is an exceedingly rare complication encountered by anesthesiologists, with its occurrence reported in the literature ranging from a mere 0.01% to potentially as high as 0.7% [5]. There are no reviews regarding its occurrence during the intraoperative period. However, among anesthetized patients, hypotension unresponsive to fluid administration is considered the primary indicator of perioperative adrenal crisis. Additional signs may only become apparent through blood analysis, including findings such as hypoglycemia, hyponatremia, and hyperkalemia [6]. Various factors can precipitate adrenal crisis during the perioperative period, including infections (bacterial, viral, mycobacterial, fungal, or parasitic), trauma, pregnancy, childbirth, anesthesia, significant emotional distress, non-compliance or abrupt discontinuation of glucocorticoid replacement therapy, and medications such as ketoconazole, etomidate, and fluconazole [7].

During major surgery under general anesthesia (including cesarean section), intraoperative steroid replacement is started at induction following the same recommendations. If recovery is uncomplicated, it is suggested to double the regular oral replacement dose of hydrocortisone for 48h or for up to a week before the maintenance dose is resumed [2].

There are no recommendations regarding additional corticosteroid administration in the event of an additional stressor, such as the need for an emergent cesarean section under general anesthesia in a patient already under supplementation for labor.

## Case presentation

**Figure 1:**
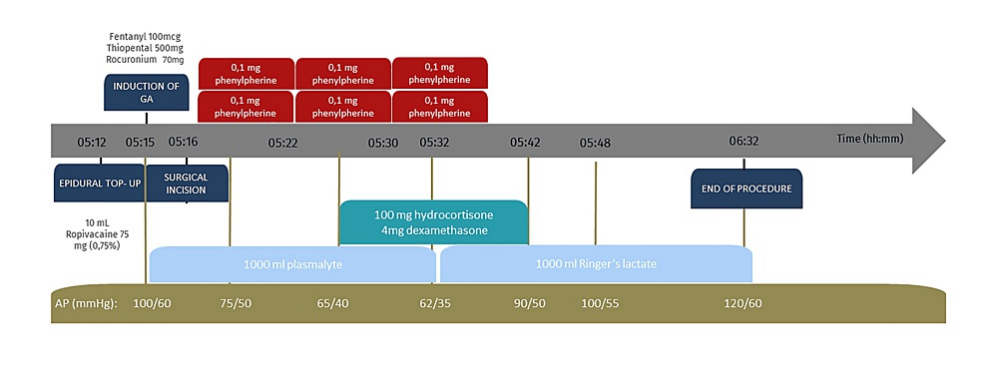
Chronologic Summary of Intraoperative Events and Interventions GA - General Anesthesia; AP: Arterial Pressure

A 26-year-old primipara with an uneventful term pregnancy was admitted to the emergency department in latent labor. Her past medical history included primary AI due to the classical form of CAH, medicated with 25 mg hydrocortisone daily. For labor analgesia, an epidural catheter was placed during the latent phase and analgesia was performed with a bolus of 12 mL of ropivacaine 0.167% with 10 mcg sufentanil followed by patient-controlled epidural analgesia with basal perfusion of ropivacaine 0.1% and sufentanil 0.25 mcg/mL. For adrenal crisis prevention, a bolus of 100 mg hydrocortisone was given at the onset of active labor, followed by 50 mg intravenous (IV) hydrocortisone every 6 hours. Fetal decelerations developed 14 hours later, and an emergency C-section was indicated. Epidural top-up was tried with 10 mL of opivacaine 0.75%, however, insufficient block height led to conversion to general anesthesia. Figure 1 presents a chronologic summary of intraoperative events and interventions.

Rapid sequence induction was performed with 500 mg thiopental and 100 mg rocuronium. The last bolus of hydrocortisone had been given 2 hours prior to the surgical incision. At the start of surgery, persistent hypotension emerged, which was refractory to fluids (2000 ml crystalloids) and a total of 600 mcg of phenylephrine. The diagnosis of AC was considered, due to which an additional bolus (100 mg) hydrocortisone was administered with sustained improvement of hemodynamic profile. The C-section was uneventful and intravenous hydrocortisone was administered as a bolus of 50 mg every 6 hours for 24 hours and then tapered to a pre-labor dose over 3 days, with no further complications. Our patient had sustained hemodynamic stability and no further complications emerged, such as organ dysfunction, hypoglycemia, or severe electrolyte disturbances. Hence, the patient remained under surveillance for a prolonged recovery at the postanesthetic care unit and later, was transferred to the obstetric ward. The newborn had an Apgar score of 5/9/9 and due to inefficient respiratory pattern and bradycardia, there was a need for positive pressure ventilation and admission to intensive care for monitoring for 5 days. Both patients were discharged with no further complications and clinical follow-up was performed by endocrinology in our hospital.

## Discussion

Our case describes a patient who probably developed acute adrenal insufficiency in the intraoperative period of a cesarean section under general anesthesia.

AC is a life-threatening emergency with a high mortality rate that results from an acute insufficiency of adrenal hormones (glucocorticoid or mineralocorticoid) [1]. It occurs most often in patients with known adrenal insufficiency who experience an acute stress event, and fail to adjust glucocorticoid therapy, culminating in the depletion of cortisol stores and leading to an Addisonian crisis [1]. It is characterized by hemodynamic instability and cardiovascular collapse, and the diagnosis requires a high index of suspicion. This situation can lead to significant morbidity and mortality and requires prompt recognition and treatment as a delay in diagnosis and management can lead to disastrous consequences.

Referred to as “the great mimic”, acute adrenal insufficiency can present with nonspecific and subtle signs and symptoms, and the diagnosis may not be apparent as clinical manifestations can be attributed to other factors present in the perioperative setting.

In our case, the diagnosis was suspected under general anesthesia. In patients under anesthesia, hypotension, which does not respond to fluid administration, has been considered the most important sign of perioperative adrenal crisis [8]. In this particular setting, with the impossibility of observing associated and often earlier symptoms, namely nausea, vomiting, abdominal pain, or altered state of consciousness, and also given the fact that stress dose supplementation was administered according to guidelines, the diagnosis was more challenging. Also, the cardinal sign of acute adrenal insufficiency, volume-resistant hypotension, can be multifactorial in this setting.

Differential diagnoses are broad and include various etiologies of shock, and cardiac conditions such as acute myocardial infarction, endocrine pathology, and infection [1]. This case demonstrates the challenges of diagnosing acute adrenal insufficiency in a context where a multitude of confounding factors are present. Particularly, there was a confounding effect of drugs with hypotensive effects, such as ropivacaine and thiopental. However, the timing of hypotension, namely following surgical incision and general anesthesia, and its persistence and lack of response to fluids and vasopressors, supports the diagnosis of adrenal crisis [1]. As hypotension from anesthetic drugs is caused by vasoplegia and cardiovascular depression, normalization with vasopressor drugs and volume would be expected. Hypovolemia was unlikely because the patient was continuously monitored, with continuous fluid therapy (polyelectrolyte), and with a urinary output greater than 0.5mL/kg/h both in the delivery room and operating room. Also, there was no excessive intraoperative blood loss or laboratory values suggesting hemorrhagic shock, and there was no difficulty in ventilation suggesting other etiologies. Sepsis was also unlikely, as the patient had no fever or other signs or symptoms suggestive of infection. Thus, the previous diagnosis of CAH and prompt and sustained normalization of blood pressure following a bolus of hydrocortisone corroborated the diagnosis of an adrenal crisis.

Although corticosteroid supplementation had been provided according to recommendations for labor, refractory hypotension emerged following the additional stress of an emergent C-section under general anesthesia. There is still no specific protocol detailing the glucocorticoid stress supplementation dose required in this situation. As we observed, even under adequate perioperative steroid coverage, a new stressor such as general anesthesia or major surgery may precipitate a crisis, making it crucial that anesthesiologists consider this diagnosis.

As there is no evidence supporting the specific postoperative management following an adrenal crisis that occurs during surgery, it should be individualized for every patient. In this setting, the stress factor was eliminated at the end of the procedure, and the patient did not develop immediate complications such as organ dysfunction, hemodynamic instability, hypoglycemia, or severe electrolyte disturbances. Following a discussion with a multidisciplinary team, including an endocrinologist, it was decided that there was no need to admit the patient to an intensive care unit. There were no adverse events during her stay. 

This case was further discussed to prevent the recurrence of adrenal crises and diagnostic delays or inadequate treatment. A careful follow-up should always be carried out to provide appropriate management in the context of a new stress event such as pregnancy, acute illness, or a new surgical procedure.

## Conclusions

Patients with AI at labor are at risk for acute adrenal insufficiency even under appropriate steroid supplementation. As perioperative adrenal crisis can be life-threatening, early identification and appropriate management are crucial to prevent severe morbidity and mortality. Recognizing an event of adrenal crisis is particularly challenging in the perioperative setting since numerous confounding factors may mask its symptoms and delay the diagnosis. Even under adequate perioperative steroid coverage, a new stressor such as general anesthesia or major surgery may precipitate a crisis. With this clinical case description along with the discussion of the possible differential diagnosis, we aim to increase awareness among anesthesiologists and other healthcare professionals about the importance of considering adrenal crisis as a potential diagnosis in similar clinical scenarios. As there are no specific recommendations following an adrenal crisis, vigilance, tailored management, and a multidisciplinary approach are key in preventing complications, recurrence, and optimizing care of patients with primary adrenal insufficiency.

## References

[1] Rathbun KM, Nguyen M, Singhal M (2023). Addisonian Crisis. StatPearls [Internet].

[2] Woodcock T, Barker P, Daniel S (2020). Guidelines for the management of glucocorticoids during the peri-operative period for patients with adrenal insufficiency: Guidelines from the Association of Anaesthetists, the Royal College of Physicians and the Society for Endocrinology UK. Anaesthesia.

[3] Stjernholm YV, Nyberg A, Cardell M, Höybye C (2016). Circulating maternal cortisol levels during vaginal delivery and elective cesarean section. Arch Gynecol Obstet.

[4] Gardella B, Gritti A, Scatigno AL, Gallotti AM, Perotti F, Dominoni M (2022). Adrenal crisis during pregnancy: Case report and obstetric perspective. Front Med (Lausanne).

[5] (2023). Orphan anesthesia. https://www.orphananesthesia.eu/en/rare-diseases/published-guidelines/acute-adrenal-insufficiency/217-acute-adrenal-insufficiency/file.html.

[6] Liu MM, Reidy AB, Saatee S, Collard CD (2017). Perioperative steroid management: Approaches based on current evidence. Anesthesiology.

[7] Elshimy G, Chippa V, Kaur J, Jeong JM (2023). Adrenal Crisis. StatPearls [Internet].

[8] Seo KH (2021). Perioperative glucocorticoid management based on current evidence. Anesth Pain Med (Seoul).

